# Acute Respiratory Failure Due to Inaugural Myasthenia Crisis

**DOI:** 10.7759/cureus.68090

**Published:** 2024-08-29

**Authors:** Irene N Ntawuruhunga, Gervais Nougon

**Affiliations:** 1 Emergency Medicine, La Clinique Saint-Luc Bouge (SLBO), Namur, BEL

**Keywords:** plasmapheresis, intravenous immunoglobulin, mechanical ventilation, respiratory failure, myasthenic crisis

## Abstract

Myasthenia gravis is a rare disease that can lead to a serious condition known as myasthenic crisis (MC). The diagnosis of MC is clinical and relies on the presence of typical symptoms that can be absent, emphasizing the importance of attracting the attention of emergency physicians to this rare cause of respiratory failure. We present the case of a 66-year-old woman presenting to the emergency department with a history of recent muscle fatigue and dehydration who developed acute respiratory failure requiring mechanical ventilation.

## Introduction

Myasthenia gravis (MG) is a rare disease with an overall prevalence of 10 to 35 per 100,000 inhabitants (0.84 to 33 cases per 100,000 inhabitants in Europe) [1,2,3]. It is characterized by fluctuating muscle weakness in the limbs, eyes, bulbar muscles, and respiratory muscles. Exacerbations of myasthenia gravis, also called myasthenia crisis (MC), occur in 15 to 30% of cases, generally within the first two to three years of diagnosis, and result from weakness of the respiratory muscles and upper airway muscles, leading to difficulty swallowing and breathing [3]. Patients experiencing myasthenia gravis may present with hypercapnic respiratory failure requiring invasive ventilatory support [3].

Acute respiratory failure is a frequent reason for consultation in emergency departments. It is defined as a dysfunction of the respiratory system leading to difficulties in maintaining adequate gas exchanges through a deficit of oxygenation (type I respiratory failure) and/or a deficit of CO2 clearance (type II respiratory failure). The most frequently encountered causes are exacerbations of asthma or chronic obstructive pulmonary disease (COPD), acute pulmonary edema, pulmonary embolism, and acute respiratory distress syndrome (ARDS) [4,5].

We report here the case of a 66-year-old female patient who was admitted to the emergency department with acute respiratory failure requiring non-invasive ventilation (NIV). The diagnosis of MC was made during her stay in intensive care. This article reviews the management of a myasthenia crisis in the emergency room.

## Case presentation

A 66-year-old female patient was admitted to the emergency room in June 2023 for progressive dehydration and dyspnea that started at least four weeks prior. The patient was apathetic but conscious, and she was brought to the department by her daughter, who explained that her mother had been hospitalized in another hospital three weeks prior for dyspnea. The assessment made during her hospitalization did not explain the origin of this dyspnea. Since her release from hospitalization two weeks prior, the patient had no longer been eating or hydrating due to a mycosis treated by fluconazole for a week by her general practitioner. The daughter felt that her mother often choked when swallowing. She explained that the patient had been very weak lately; she was regularly out of breath, she could barely stand upright, and she had difficulty keeping her head up. Her mother had been declining for a month and a half, and although she had been totally independent previously, the daughter struggled to recognize her mother in this state.

**Figure 1:**
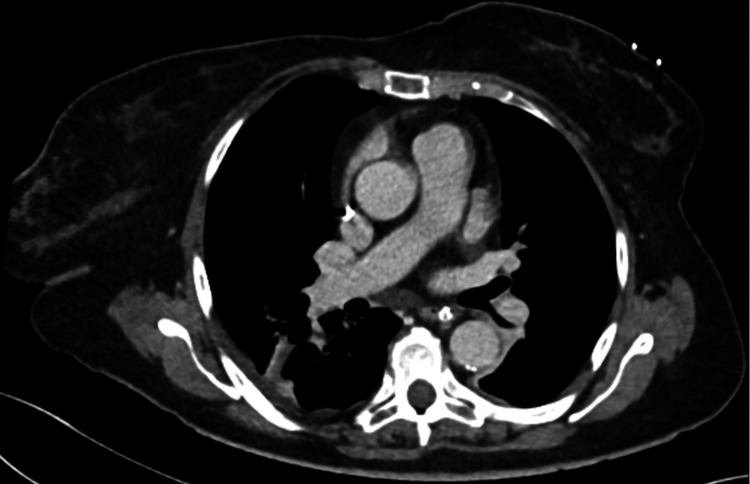
Chest CT angiogram in previous hospital

The assessment made during her previous pulmonology hospitalization in this other hospital for dyspnea of undetermined origin concluded that she had a restrictive ventilatory disorder. The possibility of pulmonary embolism was excluded by a chest CT angiogram. There was no mention of thymus residue in this chest CT (Figure 1). The patient also presented indirect signs of pulmonary arterial hypertension (PAPS 39 mmHg) on the transthoracic ultrasound. This finding was not confirmed by another transthoracic heart ultrasound that was made in our center. No further investigation was needed.

She returned with treatment by inhaled bronchodilators and magnesium in addition to her usual treatment (metformine 850 mg, L-thyroxine 50 mcg, amlodipine/valsartan 10/160 mg, gliclazide 60 mg, atorvastatin 10 mg, bisoprolol 10 mg). The rest of the outpatient assessment included a polysomnography prescription and follow-up in a cardiology and pneumonology consultation.

It can be noted that the patient's history includes type II diabetes on oral antidiabetics, treated high blood pressure, substituted hypothyroidism, and a history of intraductal carcinoma of the left breast operated in 2010. She has no known addictions or allergies.

On admission to our emergency department, the patient’s parameters showed an oxygen saturation of 93%, respiratory rate (RF) at 16/min, heart rate (HR) at 108 bpm, blood pressure at 127/83 mmHg, and a temperature of 36.8°C. She is 1.57 m tall and weighs 76 kg, which gives a body mass index (BMI) of 31.3.

The clinical examination showed a lazy skin fold, very dry oral mucous membranes, and signs of mycotic infection. The patient also exhibited diffuse and profuse sweating.

The neurological examination did not reveal any significant deficit apart from a slight paresis in the left upper limb with strength evaluated at 3/5 on the Muscle Power Scale.

Upon auscultation, heart sounds were audible without murmurs. There was a reduction in thoracic expansion. The patient’s abdomen was supple, depressible, and painless, with preserved peristalsis.

The initial assessment made in the emergency room showed acute renal failure AKIN I, probably of pre-renal origin (urea 152, creatinine 1.4, glomerular filtration rate 37), with dehydration (urea/creatinine ratio > 45) and hypernatremia (sodium 150 mEq/L) (Table 1). Gas exchanges were normal when the patient arrived (blood gas: pH 7.38, pCO2 41 mmHg, pO2 71 mmHg, bicarbonate 22.1 mmol/L, BE 3.5 mmol/L, lactate 10 mg/dL). There was no evidence of pulmonary infiltrates on the chest x-ray. The non-contrast brain scan did not show any acute ischemic or hemorrhagic lesions.

**Table 1:** Laboratory results MDRD: modification of diet in renal disease

Test	Results (normes)
Hemoglobin	14.3 gr% ( 12.0 -16.0 )
White blood cells	13 500/mm3 ( 4000 -10000 )
Absolute neutrophils	10 350/mm3 ( 2000 -7500 )
Absolute lymphocytes	1700/mm3 ( 1000 -4000 )
Urea	152 mgr% ( 19 -50 )
Creatinine	1,4 mgr% ( 0.3 -0.9 )
Glomerular filtration rate (MDRD Caucasian)	37 mL/min ( at least 60 )
Sodium	150mEq/l (135 -145 )

The patient spent the night under surveillance in the emergency room and had an episode of acute respiratory distress after drinking a glass of water. She desaturated up to 30% with bradypnea (respiratory rate 12/min); she maintained a sinus heart rhythm. The patient was immediately ventilated with a bag-mask with a fraction of inspired oxygen (FiO2) of 100%. Saturation returned quickly, and she remained conscious. The decision was made to instead attempt non-invasive ventilation (NIV). The patient was ventilated in spontaneous positive expiratory pressure-volume (SPEPV) with an AI of 8 and positive end-expiratory pressure (PEEP) at 5 cm of H20. The FiO2 was titrated for a SpO2 > 94%. A blood gas analysis showed respiratory acidosis with a pH of 7.08, a PCO2 of 73 mmHg, a PaO2 of 92 mmHg, and bicarbonates of 17.3 mmol/L. The BE value was -9.6 mmol/L. After two hours of NIV, the patient was more awake and said she was uncomfortable with the NIV. The NIV was stopped, and a blood gas count was performed again in ambient air: pH 7.21; PCO2 54 mmHg; and PaO2 69 mmHg.

An hour later, the patient’s state deteriorated again; she was drowsy and desaturated with an oxygen saturation of 88% and an RF of 12/min. She was again ventilated with NIV and transferred to the intensive care unit for treatment of hypercapnic respiratory failure of undetermined origin requiring ventilatory support by NIV. In view of the symptoms and the evolution, myasthenia gravis was suspected.

The patient was intubated after recurrence of carbonarcosis during a third attempt at weaning off the NIV. Electromyography (EMG) was performed in intensive care after five days of ventilation and showed an increase in the distal latency of the sensory potential and a discreet decrease in its amplitude of the median nerve G (Table 2), with slowing of the sensory conduction speed. Repetitive stimulation of the nerve-muscle couples: radial nerve (anconeus) and axillary nerve (deltoid nerve) highlighted a decrement estimated at a maximum of 55%, which suggested the diagnosis of myasthenia gravis. With the dosage of anti-muscle-specific kinase (anti-MUSK) and anti-acetylcholine receptor (AChR) antibodies, which came back positive, the diagnosis of respiratory failure in an initial myasthenia crisis was retained.

**Table 2:** Repetitive nerve stimulation

Nerve/Sites	Rec. Site	Onset Lat ms	Peak Lat ms	Amp µV	Segments	Distance mm	Peak Diff ms	Velocity m/s
G Median – Digit II (Antidromic)								
Wrist	Dig II	3	4	19	Wrist - Dig II	130		52
Elbow	Dig II	7	7	5	Elbow - Wrist	240	4	59
G Cubital -Digit V (Antidromic)								
Wrist	Dig V	2.2	2.9	17,3	Wrist - Dig V	110		49

On the advice of neurologists, treatment with pyridostigmine (90 mg/day, then 180 mg/day) was initiated. After 72 hours, treatment with oral corticosteroids (methylprednisolone 32 mg) was added. A progressive improvement of the respiratory symptoms and dysphagia made it possible to remove the breathing tube.

**Figure 2:**
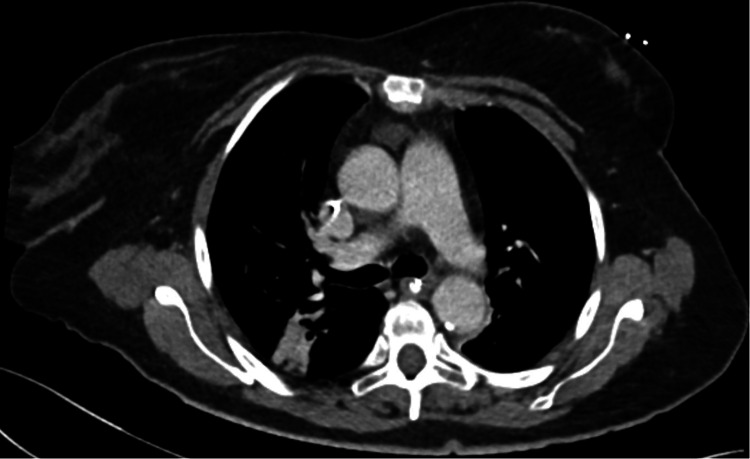
Chest CT to exclude thymoma

The search for thymoma by chest CT revealed a cystic residue of the thymus and posterior atelectasis (Figure 2). Contraindicated medications such as magnesium and beta-blockers were stopped. The patient was transferred to neurology for further treatment.

## Discussion

The case concerns a patient who presented with severe acute respiratory failure following an initial myasthenia crisis. Myasthenia gravis is the most common autoimmune disease affecting the neuromuscular junction. All races and ages are affected by myasthenia gravis, but it is most often found in young women under 40 and men over 60 [1]. The exact cause of myasthenia gravis remains undetermined. In 10 to 15% of cases, a thymoma or a thymic tumor can be found, and 3% to 8% of myasthenic patients have an autoimmune disease of the thyroid. Some authors have spoken of autoimmunity following viral infection such as the Epstein-Barr virus and SARS-CoV-2 (COVID-19) [6].

Myasthenia gravis is characterized by the destruction and/or dysfunction of postsynaptic components of the neuromuscular junction by autoantibodies, leading to a blockage of the transmission of nerve impulses. In the case described, the patient initially suffered from breathing difficulties, which led to her hospitalization for a week in another hospital. As dyspnea is not a classic mode of presentation, a diagnosis of myasthenia gravis was not made at that time. It was later that the patient developed fatigue in the head extensors, with a “drooping head” at the end of the day, as well as dysphagia. Indeed, the symptoms of the affected muscles fluctuate throughout the day, with greater fatigue at the end of the day or after exercise [3,7]. Myasthenia gravis presents with ocular symptoms in more than 50% of cases. Patients typically present with ptosis, ophthalmoplegia, or diplopia. These symptoms are often asymmetrical, and approximately 80% of these patients will progress to a generalized form within two to three years [8]. Bulbar muscle involvement is the second mode of presentation, with approximately 15% of patients presenting with dysphagia and dysarthria, but also involvement of the muscles of facial expression. This patient did not present any ocular symptoms upon admission to the emergency room or during her first stay in our institution, but she subsequently developed them during her second stay for myasthenia crisis. This is consistent with what other authors have reported in the literature regarding disease progression [7,8].

When the patient presented to our emergency department, she was not in respiratory distress; she mainly presented asthenia and dehydration during a heatwave. She went into sudden respiratory distress after asking for a glass of water and probably choking again. She arrived in the afternoon and probably became tired during her stay in the emergency room, precipitating a myasthenia crisis.

Indeed, myasthenia crisis results from an exacerbation of weakness in the muscles of the upper airways and respiratory muscles, leading to respiratory failure, which may require ventilatory support. It is either spontaneous during the active phase of the disease or precipitated by various factors, including infections, aspiration pneumonia, medications (Table 3), pregnancy, surgery, or even emotional stress. Infections account for more than 30% of myasthenia crises, with pulmonary infections at the top of the list [2,3]. The patient described in this case combined several contributing factors: she had an oral mycosis, she had hypernatremia in a context of dehydration, and she had been taking magnesium since leaving the hospital, as well as beta-blockers and benzodiazepines, which are also medications that can precipitate a myasthenia crisis.

**Table 3:** Medications likely to precipitate a myasthenia crisis Data from sources [3,9]

Antibiotics	Aminoglycosides, Quinolones, Macrolides
Antimalarials	Hydroxychloroquine
Anticonvulsants	Phenytoin, carbamazepine
Cardiovascular drugs	Beta-blockers, anti-calcium medications, class I antiarrhythmic (lidocaine and procainamide), statins
Muscle blockers	Succinylcholine
Muscle relaxants	Long-acting benzodiazepine, baclofen
Others	Iodised contrast agent, magnesium citrate

A myasthenia crisis is above all a clinical diagnosis that is more obvious to make when the diagnosis of myasthenia gravis is already known. It is an emergency because nearly 60% of patients in crisis will require invasive ventilatory support in the hours or days that follow [10]. Mortality has decreased with the evolution of treatments and is currently 4% to 8% [11].

The clinical examination must make it possible to differentiate between myasthenia and other neuromuscular pathologies such as cholinergic crisis, Guillain-Barré syndrome, Lambert Eaton syndrome or pseudo-myasthenia, myelopathy, polymyositis, or toxic causes (botulism, organophosphates, etc.).

If ptosis is present, an ice test can confirm the diagnosis of myasthenia gravis. This involves applying ice cubes to the eye affected by ptosis or ophthalmoparesis for two minutes and seeing if there is an improvement of more than 2 mm in the opening of the eye. This test makes it possible to diagnose a myasthenia crisis with a sensitivity of 92% and a specificity of 72% [3].

Clinical examination of the respiratory system can be misleading because some patients in a state of myasthenia gravis present “silent respiratory distress” with auscultatory hypoventilation but without the use of accessory muscles. The single breath test, which involves asking the patient to take a deep breath and count out loud in a single exhalation until they can no longer speak, is a simple way to assess respiratory function. A threshold of 15-20 words is considered an indication to start ventilatory support. The edrophonium test is no longer used due to its toxicity and poor performance in diagnosing myasthenia gravis.

This patient's myasthenia crisis caused respiratory failure with carbonarcosis, requiring non-invasive ventilatory support in the emergency room. For patients who maintain a level of consciousness that allows them to protect their airways, NIV has shown a reduction in the duration of ventilatory support as well as a reduction in the length of stay in the intensive care unit [12]. The patient improved clinically, and her gas exchange improved under NIV, but it quickly deteriorated again after stopping the NIV, showing significant muscle fatigue. Intubation could have been suggested in the emergency room given this clinical picture. Some authors have proposed PCO2 thresholds of 45-50 mmHg as an indicator of non-invasive ventilation failure [12,13]. Indeed, the blood gas count performed after one hour of mechanical ventilation showed a PCO2 of 54 mmHg. The patient was put back on NIV and transferred to intensive care, where she was intubated after a second attempt at weaning off NIV. There are no special considerations for intubating a patient with a myasthenia crisis other than the medications to be used. Myasthenic patients are less sensitive to depolarizing muscle relaxants such as succinylcholine, which should be avoided [14]. For non-depolarizing muscle blockers, doses lower than standard doses should be used. Roper et al. suggest reducing doses by half or a third for patients with myasthenic crises [3]. It is also possible to perform intubation without muscle blockers.

The diagnosis of myasthenia gravis was confirmed by electromyography performed in intensive care, and the search for anti-AChR antibodies came back positive, as in the majority of cases in patients with generalized myasthenia gravis. Indeed, approximately 85% of patients with generalized myasthenia gravis have anti-AChR antibodies, 8% have anti-muscle-specific kinase (anti-MUSK) antibodies, and 1% have anti-low-density lipoprotein receptor-related protein 4 (LRP4) antibodies. Patients are said to be seronegative if none of these antibodies are found [15].

Per the opinion of the neurologists, treatment with pyridostigmine 90 mg/day and then 180 mg/day was started when electromyography confirmed myasthenia gravis, i.e., five days after the patient arrived in intensive care. Oral corticosteroids were started after 72 hours at a dose of 32 mg of methylprednisolone. Corticosteroids should be avoided in the emergency room because they can worsen the myasthenia crisis. Pyridostigmine should also be avoided in myasthenia crises because it causes heart rhythm disturbances and an increase in secretions, which can compromise breathing [11,16]. The IV immunoglobulin (1-2 g/kg) and plasmapheresis constitute the “gold standard” for the treatment of severe myasthenia crises requiring ventilatory support. Only immunoglobulins can be initiated in the emergency room [3,17]. Plasmapheresis is not performed in our center.

There was a delay in initiating treatment for the myasthenia gravis of this patient due to the atypical presentation of her disease. Although myasthenia gravis is a clinical diagnosis, this case showed its rare mode of presentation. Additionally, the patient did not have ocular symptoms that are common in myasthenia gravis. Immunoglobulin treatment could have been initiated upon arrival in intensive care and would perhaps have made it possible to reduce the length of stay in intensive care under invasive ventilatory support. However, there is not enough data in the literature to decide on this question, as shown by Mario B. Prado Jr. et al. in a systematic review comparing acetylcholine esterase inhibitors to IV immunoglobulins and/or plasmapheresis [18]. Nevertheless, the patient's evolution was favorable, and she was able to be weaned from invasive ventilation 72 hours after the initiation of treatment without any other form of complication.

## Conclusions

Myasthenia gravis can present as respiratory distress and should be considered when faced with respiratory distress associating neuromuscular symptoms. Ocular symptoms, although often present and pathognomonic, may be absent. Respiratory failure can be insidious to start with and can deteriorate rapidly. A myasthenia crisis is a clinical diagnosis, and treatment should not wait for the results of diagnostic tests. The standard treatment for a myasthenia crisis is ventilatory support (invasive or not) and immunoglobulins and/or plasmapheresis.
